# Increasing Antioxidant Activity in Food Waste Extracts by β-Glucosidase

**DOI:** 10.17113/ftb.60.04.22.7443

**Published:** 2022-12

**Authors:** Farahnaz Karami, Mohammad Ghorbani, Alireza Sadeghi Mahoonak, Alireza Pourhossein, Ahmad Bagheri, Reza Khodarahmi

**Affiliations:** 1 Department of Food Science and Technology, Gorgan University of Agricultural Sciences and Natural Resources, Basij street, 4918943464 Gorgan, Iran; 2 Medical Biology Research Center, Kermanshah University of Medical Sciences, Daneshgah street, 6714415185 Kermanshah, Iran; 3 Nano Drug Delivery Research Center, Health Technology Institue, Kermanshah University of Medical Sciences, Daneshgah street, 6714415153 Kermanshah, Iran; 4 Department of Pharmacognosy and Biotechnology, Faculty of Pharmacy, Kermanshah University of Medical Sciences, Daneshgah street, 6714415153 Kermanshah, Iran

**Keywords:** antioxidant activity, β-glucosidase, oleuropein, quercetin, food waste

## Abstract

**Research background:**

Food by-products such as onion peels and olive leaves are rich in bioactive compounds applicable as natural and low-cost sources of antioxidants. Still, these compounds mainly exist in glycosylated form. Often, hydrolysis of glycoside compounds increases their antioxidant activity and health benefits. However, not many studies have been done concerning the β-glucosidase effect, specifically from *Aspergillus niger*, on glycosylated compounds within these by-products. Also, changes in the antioxidant activity of the mentioned by-products under the effect of β-glucosidase have not been reported yet. Therefore, this study considers the effect of *A. niger* β-glucosidase on glucoside compounds and the antioxidant activity of onion peel and olive leaf extracts.

**Experimental approach:**

The antioxidant activity of the extracts was determined by 1,1-diphenyl-2-picrylhydrazyl (DPPH) and ferric reducing antioxidant power (FRAP) assays. Also, glucose, total phenolic and flavonoid contents were measured. Moreover, TLC and HPLC analyses were performed before and after the enzymatic hydrolysis.

**Results and conclusions:**

The obtained results showed an increase in the extract antioxidant activity after treatment. Also, β-glucosidase increased the glucose content of the extracts. The thin layer chromatography (TLC) and high-performance liquid chromatography (HPLC) results showed the β-glucosidase efficacy to hydrolyze quercetin glucosides in onion peel extract, and the quercetin concentration increased from (0.48±0.04) mg/mL in the untreated extract to (1.26±0.03) mg/mL in the treated extract (0.5% *m*/*V*) after 3 h of enzymatic hydrolysis at 45 °C. Also, the content of quercetin-3-O-glucoside increased considerably from (1.8±0.1) to (54±9) µg/mL following the enzyme treatment. Moreover, oleuropein in olive leaf extract (1% *m*/*V*) was hydrolyzed completely from (0.382±0.016) to 0 mg/mL by β-glucosidase for 24 h at 50 °C.

**Novelty and scientific contribution:**

This study showed that *A. niger* β-glucosidase, as a stable enzyme, hydrolyzed quercetin and oleuropein glycosides in onion peel and olive leaf extracts. Thus, *A. niger* β-glucosidase is a good candidate for processing the food waste and extracting valuable bioactive compounds. Also, the treated extracts with higher antioxidant and biological activity, and without bitter taste can be applicable as potent, natural and cost-effective antioxidants in the food industry.

## INTRODUCTION

Due to increasing awareness about health benefits of fruits and vegetables, their consumption has increased. Nevertheless, many fruits and vegetables are processed to extract the valuable ingredients. During the processing of these crops, enormous amounts of waste are generated. The resulting by-products from plant processing are generally used as raw materials for animal feed or agricultural uses. They are rich in bioactive components applicable in different industries as the natural, economical, novel and low-cost sources of antioxidants, dietary fibre, organic acids, enzymes, pectin, food additives, essential oils, *etc.* (*1*).

The onion peel is one of these by-products. The outer scales, peel and onion roots are removed and regarded as waste during food processing. Various onions (white, yellow and red) are rich in flavonols such as quercetin (mainly), kaempferol, tannins and organosulfur compounds. Quercetin, quercetin-4-O-glucoside and quercetin-3,4-O-diglucoside are major forms of quercetin in onions (*2*). Quercetin is a food additive, colourant and raw material for the pharmaceutical, cosmetic and fine chemical industries (*3*). The onion peel contains more quercetin derivatives than the onion bulb scale and other vegetables and fruits. Gorinstein *et al*. (*4*) declared that quercetin in onion peel was 48-fold higher than in the bulb scale. Therefore, the onion peel is an economic source of bioactive compounds.

Also, olive leaves are inexpensive by-products of olive trees and olive oil mills. Oleuropein is the main phenolic compound in olive leaves and fruit and an agent of bitterness in olives. It is an ester of hydroxytyrosol, elenolic acid and glucose (*5*), which after hydrolysis gives oleuropein aglycone with antioxidant, bacteriostatic, anticancer, and antiviral properties (*6*). Also, hydrolysis of oleuropein aglycon produces hydroxytyrosol, a potent antioxidant without bitter taste. The production of these valuable compounds from renewable materials makes olive waste reusable.

Although the by-products obtained from fruit and vegetable processing contain phenolic and flavonoid compounds with high antioxidant activity, these compounds mainly exist in glycosylated form. Often, the nonglycosylated form of these substances has been shown to have higher antioxidant activity than their glycosides. Also, hydrolysis of glycosylated components increases the extraction yield of their aglycones. Moreover, enzymatic hydrolysis is preferred to chemical one because the enzyme selectively acts on desired compounds, produces fewer secondary metabolites, other valuable compounds are not damaged, and reduces environmental problems. Thus, enzymatic hydrolysis can be an alternative to chemical methods.

β-Glucosidases (β-d-glucoside glucohydrolase, EC 3.2.1.21) are biological macromolecules. These enzymes break glycosidic bonds in alkyl- and aryl-β-d-glycosides and various oligosaccharides (*7*). One of the technological applications of β-glucosidases is the release of phenolic compounds (in aglycone form) with antioxidant activity from fruit and vegetable residues. In this manner, *Pyrococcus furiosus* β-glucosidase has been used for hydrolysis of flavanone glycosides in grapefruit pulp, and grapefruit and orange peel extracts (*8*). The recombinant thermostable β-glucosidases from *Thermotoga neapolitana* convert quercetin glucosides to quercetin in onion waste extract (*9*). The recombinant β-glucosidase from *Myceliophthora thermophila* and β-glucosidases from almonds hydrolyze oleuropein from olive leaf extracts (*10*, *11*). Moreover, β-glucosidase from *Aspergillus niger* hydrolyzes this compound in olive mill wastewater (*12*).

However, few studies have been done concerning the β-glucosidase hydrolysis of glycosylated compounds in onion peel and olive leaf extracts, specifically β-glucosidase obtained from *A. niger* (as a food-grade and inexpensive microorganism for enzyme production with potential application on an industrial scale). Moreover, the effect of this enzyme on the antioxidant activity of the mentioned by-products has not been reported. This study investigated the efficacy of β-glucosidase from *A. niger* on glycoside components and antioxidant activity of onion peel and olive leaf extracts due to valuable ingredients in these food residues for potential application in food as natural and cost-effective antioxidants. The effect of β-glucosidase amounts and reaction time on the antioxidant activity of the onion peel and olive leaf extracts was measured using DPPH and FRAP tests. Also, the enzyme efficacy for hydrolysis of glycoside compounds was determined by measuring the concentration of glucose in the extract and conducting TLC and HPLC analyses before and after the enzyme treatment.

## MATERIALS AND METHODS

### Materials

*Aspergillus niger* β-glucosidase from previous work was used (*13*). The fungus was isolated from farm soil, and its genus and species were determined before application. The β-glucosidase was purified in two stages of ion-exchange chromatography. Onion was purchased from a local market (Elaheyeh, Iran). Olive leaves were separated from olive trees in local orchards in Kermanshah province, Iran. Quercetin, quercetin-3-O-glucoside, Trolox and 1,1-diphenyl-2-picrylhydrazyl (DPPH) were purchased from Sigma-Aldrich, Merck (St. Louis, MO, USA). Oleuropein was supplied from Cayman Chemical Company (Ann Arbor, MI, USA). Other chemical materials were of analytical grade and obtained from Merck (Darmstadt, Germany).

### Extract preparation

Onion peel extract was prepared according to Lee *et al*. (*14*) with modifications. At first, aqueous ethanol (20 mL of 60% solution) was added to 1 g of dried and powdered red onion peel, and the mixture was stirred at room temperature for 60 min. Then, the mixture was filtered through a Büchner funnel with Whatman No. 2 filter paper, and again 20 mL of solvent were added to the residue. Extraction was repeated for another 30 min. After filtration, the solvent was removed at 40 °C by a rotary evaporator (SB-1200; EYELA, Tokyo, Japan) under a vacuum.

Olive leaf extract was prepared according to Yateem *et al*. (*15*) with modifications. Primarily, olive leaves were washed, dried in an oven at 50 °C and powdered. Then, 100 mL of 80% aqueous ethanol were added to 10 g of olive leaf powder, and the mixture was stirred for 4 h at room temperature. After filtration through the Whatman filter paper No. 1, ethanol was evaporated at 40 °C by a rotary evaporator (SB-1200; EYELA) under a vacuum. Then, 15 mL of water was added to the extract, and it was centrifuged (2-16PK; Sigma-Aldrich, Merck, Berlin, Germany) at 4724×*g* for 5 min to separate chlorophyll and other water-insoluble compounds. Next, 2 mL of dichloromethane were added to the extract, and after mixing for 1 min, the mixture was centrifuged. Finally, the water was evaporated as described in the previous section and the extracts were stored at 4 °C until analysis.

### Effect of β-glucosidase on the antioxidant activity of onion peel and olive leaf extracts

#### DPPH assay

For the DPPH radical scavenging activity assay, extract solutions of 0.02% onion peel and 0.1% olive leaf (by *m*/*V*) were prepared in 100 mM acetate buffer (pH=5). Onion peel stock solution (0.5%) was prepared in acetate buffer containing *φ*(ethanol)=30%. Then, 100 µL of β-glucosidase in the same buffer (0.1 µg/mL in reaction medium) were added to 400 µL of each extract, and the reaction was done at time intervals of 15 min for 90 min at 60 °C. Also, various enzyme concentrations (0–1 µg/mL) were reacted with the extracts in a total volume of 500 µL at 60 °C for 15 min. The enzyme was inactivated by heating the extracts at 90 °C for 5 min. DPPH assay was carried out according to Brand-Williams *et al*. (*16*) with slight modifications. DPPH stock solution was prepared by dissolving 4.8 mg of DPPH in 20 mL methanol and was stored at –20 °C until analysis. For the analysis, DPPH solution was diluted with methanol 5.5 times. Then, 950 µL of this solution reacted with 50 µL of the extract for 1 h in the dark. The blank sample was prepared by adding 50 µL of acetate buffer to 950 µL of DPPH reagent. The absorbance was read at 515 nm by a UV-Vis spectrophotometer (Lambda 25; PerkinElmer, Waltham, MA, USA) and the percentage of radical scavenging was calculated as follows:

Radical scavenging=((*A*_b_−*A*_s_)/*A*_b_)·100 /1/

where *A*_b_ and *A*_s_ are the absorbances of blank and sample, respectively.

#### Ferric reducing antioxidant power assay

The FRAP assay is based on the increase of the absorbance at 595 nm due to 2,4,6-tripyridyl-*s*-triazine (TPTZ)-Fe^2+^ complex formation. For this test, extract solutions were prepared at (by *m*/*V*) 0.05% olive leaves and 0.01% onion peel. As stated in the previous section, the effect of enzyme concentration and reaction time was assayed measuring the extract reducing power. FRAP assay was performed according to Benzie and Strain (*17*). The stock reagents, namely acetate buffer (300 mM, pH=3.6), FeCl_3_·6H_2_O solution (20 mM) and TPTZ solution (10 mM in 40 mM HCl), were prepared. The working reagent was freshly prepared by combining 2.5 mL TPTZ, 2.5 mL FeCl_3_·6H_2_O and 25 mL of acetate buffer solutions and warmed to 37 °C before the use. The extract (50 µL) was added to the working reagent (950 µL) and incubated at 37 °C for 30 min in the dark. The absorbance was read at 595 nm. The standard diagram was obtained by measuring the absorbance of Trolox standard solution in the concentration range 0–500 µM, and antioxidant activity was expressed in µM of Trolox equivalents.

### Measurement of total phenolic and flavonoid contents in onion peel and olive leaf extracts

Total phenols were measured before and after the reaction of β-glucosidase with the extracts using the Folin-Ciocalteu reagent according to Roesler *et al*. (*18*) with modifications. For this purpose, β-glucosidase was added at concentrations of 1.5 and 2 μg/mL, respectively, in a total volume of 500 µL, to 400 µL of olive leaf (1% *m*/*V*) and onion peel (0.5% *m*/*V*) extracts. The reaction time of the extracts with β-glucosidase was 24 h at 50 °C for olive leaves and 3 h at 45 °C for onion peel. Enzyme was inactivated by heating the extracts at 90 °C for 5 min. The extracts (100 µL) were mixed with Folin-Ciocalteu reagent (500 µL of 1:10 dilution), and the mixtures were incubated at 25 °C for 5 min. Then, 400 µL of 5% Na_2_CO_3_ were added, and again incubated in a water bath (ONE-29; Memmert, Schwabach, Germany) at 50 °C for 5 min. Next, the absorbance was read at 765 nm after cooling the samples to room temperature. A standard diagram was prepared using gallic acid (0–80 µg/mL) to estimate total phenolic content (TPC). The TPC was calculated in mg gallic acid equivalents per g dried powders of onion peel or olive leaves.

Total flavonoid content of the extracts before and after the addition of β-glucosidase was determined according to Kosalec *et al*. (*19*). Initially, 250 µL of the extract were poured into a test tube. Then, it was mixed with 750 µL of 96% ethanol, 50 µL of 10% AlCl_3_ (*m*/*V*), 50 µL of 1 M sodium acetate and 1.4 mL of distilled water. After the incubation for 30 min at room temperature, the absorbance was read at 415 nm. A standard diagram was provided using quercetin standard solution (0–100 µg/mL) and total flavonoid content was expressed as mg quercetin equivalents per g dried powders of onion peel or olive leaves.

### Glucose measurement

The glucose content was measured by hexokinase kit (Greiner Diagnostic GmbH, Bahlingen, Germany) before and after the enzymatic hydrolysis, which was used because the phenolic and flavonoid compounds in the onion peel and olive leaf extracts interfere with glucose in conventional methods such as the dinitrosalicylic acid (DNS) and glucose-oxidase/peroxidase assays. The effect of β-glucosidase addition to the extract was investigated as stated in the chapter above. After inactivation of the enzyme, the absorbance of the extracts with and without the enzyme treatment was read at 340 nm. The glucose concentration (mg/100 mL) was determined according to the manufacturer’s instructions.

### Thin-layer chromatography analysis

The effect of β-glucosidase addition on the investigated extracts was determined as described in the section on total phenolic and flavonoid measurements. After the enzyme inactivation, the extracts were analyzed by loading 5 μL of the reaction mixture on TLC plates (silica gel 60 F254, 20 cm×20 cm; Merck, Darmstadt, Germany). Glucose, quercetin, quercetin-3-O-glucoside and oleuropein were used as standards. The aforementioned compounds were separated by the solvent comprising *V*(butanol):*V*(acetic acid):*V*(water)=3:1:1. The spots were visualized under a UV light at 254 nm (quercetin and oleuropein) or by dipping plates in a methanol solution containing 5% (*V*/*V*) sulfuric acid and heating at 120 °C for 10 min (glucose) (*20*). The semi-quantitative analysis of the TLC plate was done using ImageJ software (*21*). The TLC plate was analyzed vertically for lines related to the extracts before and after the enzyme treatment, and the results were expressed as the percentage of peak area for desired compounds (quercetin and oleuropein). Also, the TLC plate was analyzed horizontally for a semi-quantitative comparison of glucose in the extracts before and after the treatment. The results were expressed as the percentage of glucose peak area.

### High-performance liquid chromatography analysis

The effect of β-glucosidase treatment on the extracts and oleuropein standard was investigated as described in the section of total phenolic and flavonoid measurements. Oleuropein standard was applied at a concentration of 1 mg/mL. The total volume of the reaction mixture for samples and standard was 500 µL. A volume of 500 µL of ethanol was added to each extract and standard at the end of the reaction time. Oleuropein, quercetin and quercetin 3-O-glucoside standards were prepared as 50% (*V*/*V*) ethanol solutions at the concentration of 1 mg/mL. The standards and extracts were filtered and analyzed by an Agilent 1260 HPLC system (Agilent Technologies, Santa Clara, CA, USA). The separation of onion peel extract was done using the MZ PerfectSil™ 100 ODS-3 column (250 mm×4.6 mm×5 µm; MZ-Analysentechnik GmbH, Mainz, Germany). The analysis conditions were chosen according to Kim *et al*. (*22*). The mobile phase included *V*(water):*V*(formic acid)=95:5 (A) and 100% methanol (B). The binary gradient was applied as follows: 20–60% B for 0–25 min, 60–100% B for 25–25.1 min, 100–60% B for 25.1–30 min, 60–20% B for 30–30.1 min, and 20% B for 30.1–35 min. The flow rate and the injection volume were 1 mL/min and 10 µL, respectively. The absorbance was read at 360 nm. Olive leaf extract was analyzed according to Mazzei *et al*. (*23*). The mixture of *V*(water):*V*(acetonitrile)=79:21 was acidifed with *o*-phosphoric acid up to pH=3 and used as a mobile phase in the same column. The flow rate and the injection volume were 1.2 mL/min and 5 µL, respectively. Compounds were detected at 280 nm and ingredients in the extracts were identified by comparing their retention times with standards and literature data. Also, quercetin, quercetin-3-O-glucoside and oleuropein content in the extracts were measured before and after β-glucosidase treatment using the related standard calibration diagrams in the range 0–1 mg/mL for quercetin-3-O-glucoside and oleuropein and 0–0.5 mg/mL for quercetin. Calculations were performed based on 0.5% onion peel and 1% olive leaves extracts.

### Statistical analysis

Using ANOVA, the data were analysed by SAS v. 9 software (*24*). The significant differences were considered using the LSD test at p<0.05. The semi-quantitative analysis of the TLC plate was carried out by ImageJ software ([REMOVED HYPERLINKF0 FIELD]*21*).

## RESULTS AND DISCUSSION

### Antioxidant activity of onion peel and olive leaf extracts after treatment with β-glucosidase

#### DPPH radical scavenging activity of the extracts

**Fig. 1:**
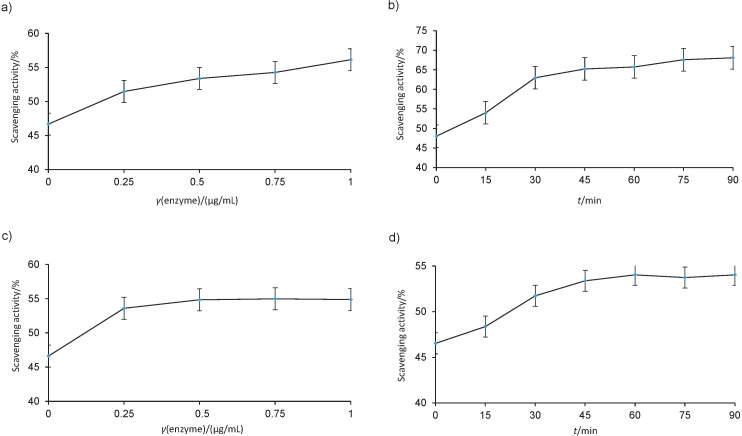
The influence of β-glucosidase concentration and reaction time on DPPH radical scavenging activity of: a and b) onion peel, and c and d) olive leaf. The data are an average of two replicates

The effect of β-glucosidase treatment on the radical scavenging activity of the extracts is shown in Fig. 1. The antioxidant activity of the extracts was enhanced with an increase in the enzyme concentration, but in olive leaf extract, the antioxidant activity remained constant after the addition of 0.25 µg/mL β-glucosidase. The extract reaction time with β-glucosidase significantly affected DPPH radical scavenging (p<0.05), and antioxidant activity increased up to 45 (onion peel) and 60 min (olive leaf), after which it remained almost constant for both extracts. At the end of 90 min of reaction with the enzyme, the DPPH radical scavenging was 68.05 and 54.02% for onion peel and olive leaf extracts, respectively, which is an increase of 1.41 and 1.16 times compared to the initial scavenging activity of non-treated extracts. This enhancement is related to the hydrolysis of glycosidic compounds by β-glucosidase in the extracts and the increase of the number of free hydroxyl groups in the flavonoid ring (*25*).

Similarly, the antioxidant activity of citrus by-products increased with tannase, pectinase, cellulase and β-glucosidase treatment (*26*). A slight decrease in the antioxidant activity was observed after the reaction of olive leaf extract with β-glucosidase at 50 °C for 24 h (data not shown) due to the hydrolysis of oleuropein aglycon in the water. Similar results were obtained by Yuan *et al*. (*27*), who observed that olive leaf extract scavenging activity decreased after hydrolysis of oleuropein by hemicellulase.

#### Antioxidant activity of the extracts determined by FRAP assay

**Fig. 2:**
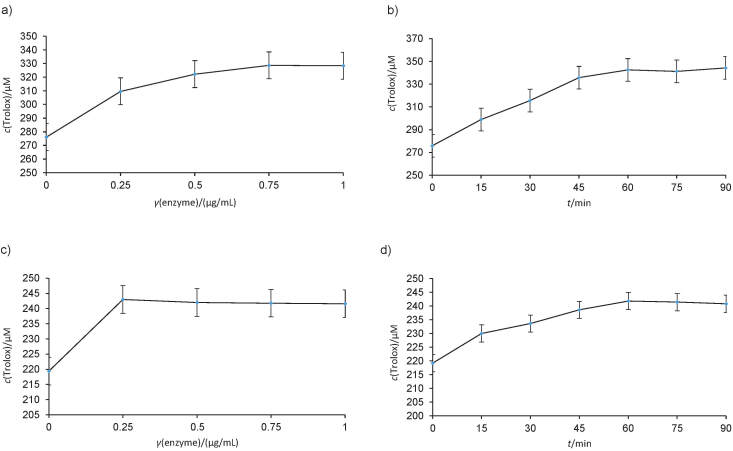
The influence of β-glucosidase concentration and reaction time on: a and b) reducing power (FRAP) of onion peel, and c and d) olive leaf. The data are an average of two replicates

The results of FRAP assay showed increased antioxidant activity expressed in µM of Trolox equivalents (TE) for both extracts after the enzyme treatment (Fig. 2). The antioxidant activity remained constant in both onion peel and olive leaf extracts at concentrations higher than 0.75 and 0.25 µg/mL of β-glucosidase. Also, the extract reaction with β-glucosidase had a significant effect on the antioxidant activity. Among the treated extracts, the highest antioxidant activity was obtained for onion peel after 90 min (from 275.86 to 344.26 µM TE), followed by olive leaves (from 219.2 to 240.8 µM TE). The obtained results for the FRAP test are in the agreement with the ones obtained by the DPPH test. Kim and Jang (*28*) observed a significant increase in the antioxidant activity of mulberry leaf extract when using β-glucosidase. They performed the antioxidant activity assay by oxygen radical absorbance capacity (ORAC) and cellular antioxidant capacity (CAC). Different results were obtained by Cavia-Saiz *et al*. (*29*) when treating grapefruit juice with naringinase. They observed lower antioxidant activity in the treated extract than in the fresh juice, while the antioxidant capacity determined by the 2,2'-azino-bis(3-ethylbenzthiazoline-6-sulphonic acid (ABTS) method was higher, which could be due to the loss of vitamin C during pasteurization of the extract and consequent decrease in the extract reducing power. In another study, antioxidant activity increased in raspberry waste after treatment with commercial cellulase, hemicellulase and pectinase. The antioxidant activity was assayed by DPPH, ABTS and FRAP tests (*30*). In a recent study, we have evaluated the effect of *A. niger* β-glucosidase on grapefruit peel extract. The data showed an increase of 2.09 times in the extract antioxidant activity after the enzymatic treatment for 90 min (*31*).

The antioxidant activity data obtained by FRAP and DPPH assays point out that more bioactive and valuable extracts were obtained after the enzymatic hydrolysis, which makes them applicable as inexpensive, robust and natural antioxidant compounds in the food industry.

### Mass fractions of total phenols and flavonoids in onion peel and olive leaf extracts

Mass fractions of total phenols and flavonoids were measured before and after the enzymatic hydrolysis, using gallic acid and quercetin as standards, respectively. Total phenolic mass fraction slightly increased from (16.1±0.2) to (17.5±0.7) mg/g in dry olive leaf extract and from (40.8±1.1) to (41.7±0.6) mg/g in dry onion peel extract after the enzymatic treatment. Also, flavonoid mass fraction increased after enzymatic treatment from (2.31±0.07) to (2.66±0.03) mg/g in the olive leaf extract, while it remained unchanged (16.3±0.5) mg/g in the onion peel extract. However, this increase was not significant (p<0.05). Total phenolic and flavonoid contents remain almost constant before and after the enzymatic treatment, because after the enzymatic hydrolysis, only the glucose is separated from the glycosylated compounds, producing their aglycon. Although their total amount in the medium is not changed, the content of each phenolic and flavonoid component may increase or decrease after the treatment.

Moreover, measuring the total phenolic content with the Folin-Ciocalteu method, higher values were obtained due to the unselectivity of the reagent. This reagent reacts with other reducing agents such as sugars (glucose) and phenolic compounds (*32*) forming blue dye with hydroxyl groups of phenols. After the enzymatic hydrolysis, blue dye intensity increases due to the separation of glucose from the glycoside compounds and an increase in free hydroxyl groups in the flavonoid ring. Cavia-Saiz *et al*. (*29*) observed a 78% increase in grapefruit juice total phenols after the treatment with naringinase. Wang *et al*. (*33*) observed a significant increase in soluble phenol and flavonoid content when treating guava leaf extract with a mixture of β-glucosidase and cellulase. In another report, the content of phenolic and flavonoid compounds in oilseed cake extract of *Borago officinalis, Oenothera biennis* and *Nigella sativa* increased after the treatment with β-glucosidase, β-glucanase, ɑ-amylase and their combinations (*25*). Ruviaro *et al*. (*26*) obtained similar data by treating citrus waste extract with tannase, pectinase, cellulase and β-glucosidase.

### Glucose concentration in the extracts

The glucose concentration increased significantly in onion peel extract from (222.2±1.2) to (247.76±0.3) mg/100 mL and in olive leaf extract from (27.0±0.7) to (75.6±0.6) mg/100 mL after the β-glucosidase treatment (2.8- and 1.11-fold for olive leaf and onion peel extracts, respectively). The high glucose concentration in olive leaf extract after enzymatic treatment is related to oleuropein hydrolysis as the main phenolic compound in olive leaves. The increase in glucose concentration in onion peel extract is associated with the hydrolysis of quercetin glucosides by β-glucosidase. Similar results were observed in the study of Ratz-Lyko *et al*. (*25*).

### Results of thin-layer chromatography analysis

**Fig. 3:**
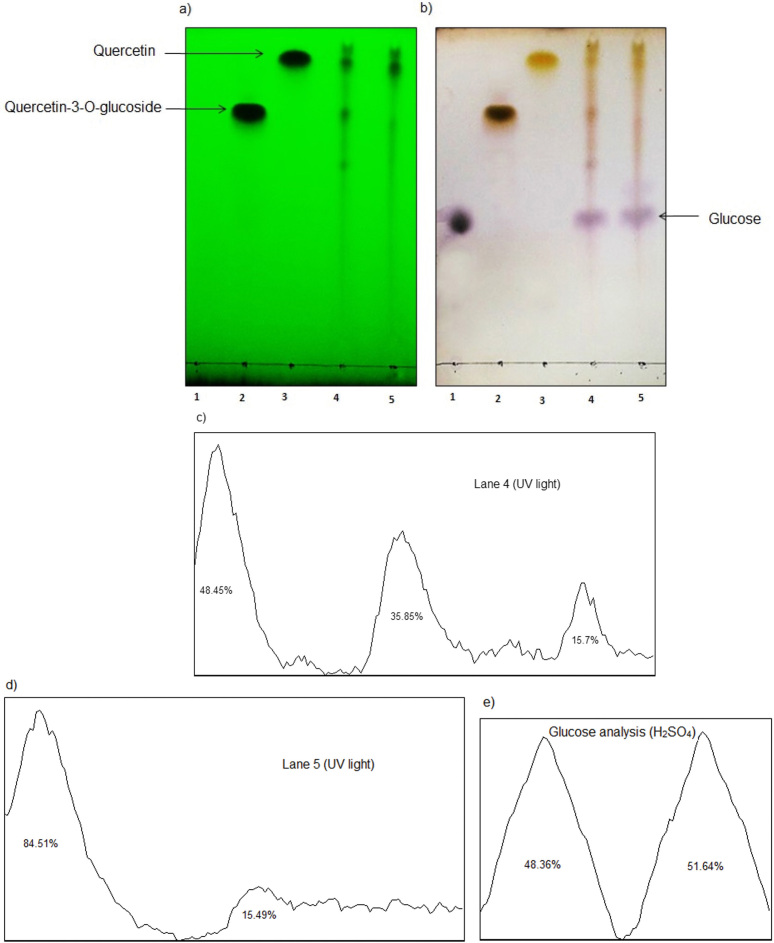
Thin-layer chromatography (TLC) analysis of the effect of β-glucosidase on glycoside compounds in onion peel extract: a) plate visualized by UV light at 254 nm, and b) treated with 5% H_2_SO_4_ methanol solution. Lanes 1-5: glucose, quercetin 3-O-glucoside, quercetin, the extract before enzymatic treatment, and the extract after the enzyme treatment, respectively, c) vertical analysis of the TLC plate (a) was done before enzymatic hydrolysis (lane 4), and d) after the enzymatic hydrolysis (lane 5) by ImageJ software (*21*), e) glucose changes on TLC plate (b) before and after the treatment were investigated by horizontal analysis of lanes 4 and 5 using ImageJ software (*21*)

**Fig. 4:**
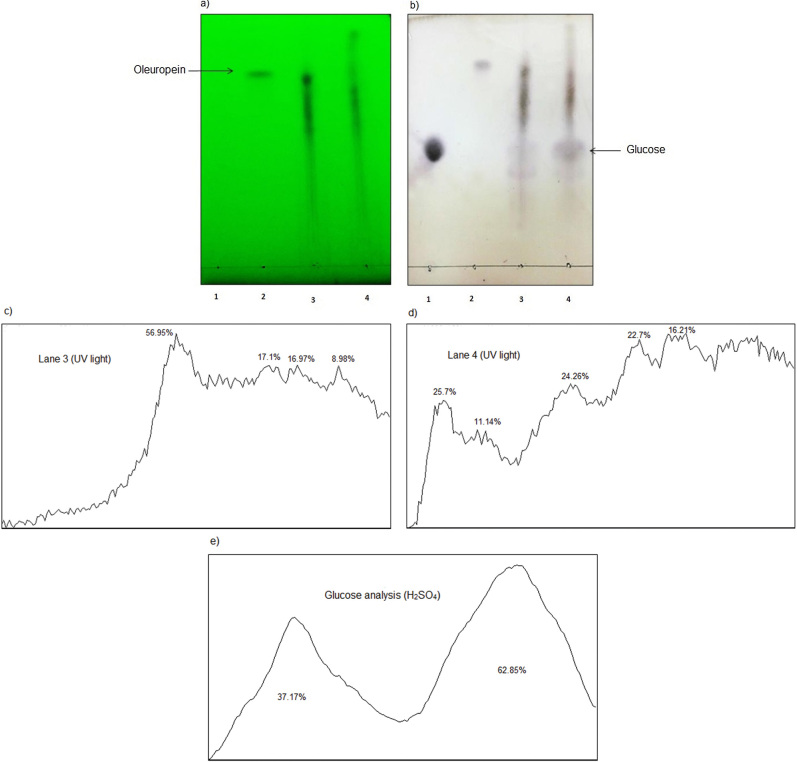
Thin-layer chromatography (TLC) analysis of the effect of β-glucosidase on glycoside compounds in olive leaf extract: a) visualized by UV light at 254 nm, and b) treated with 5% H_2_SO_4_ methanol solution. Lanes 1-4: glucose, oleuropein, the extract before the enzymatic treatment and the extract after the enzymatic treatment, respectively, c) vertical analysis of the TLC plate (a) was done before enzymatic hydrolysis (lane 3), and d) after the enzymatic hydrolysis (lane 4) using ImageJ software (*21*), e) glucose changes on TLC plate (b) before and after the treatment were investigated by horizontal analysis of lanes 3 and 4 using ImageJ software (*21*)

The results of the TLC analysis are shown in Fig. 3 and Fig. 4. According to Fig. 3a, quercetin glycosides were hydrolyzed by treating the onion peel extract with β-glucosidase. As a result, quercetin amount increased in onion peel extract after the enzymatic hydrolysis. The TLC plate analysis (Fig. 3c and 3d) confirmed the quercetin increase in onion peel extract after the enzyme action (quercetin peak area from 48.45 to 84.51%). Also, the extract treatment increased glucose amount (glucose peak area from 48.36 to 51.64%) (Fig. 3e). Choi *et al*. (*34*) obtained similar data by treating onion skin waste with cellulase, xylanase and pectinase. Also, β-glucosidase hydrolyzed oleuropein in olive leaf extract entirely (Fig. 4a), and as a result, the oleuropein was not present in the olive leaf extract after the enzymatic hydrolysis.

Moreover, glucose amount increased considerably in olive leaf extract after the enzymatic treatment (Fig. 4b). The obtained data confirmed the complete hydrolysis of oleuropein and increase of glucose (glucose peak area from 37.17 to 62.85%) in the treated olive leaf extract (Fig. 4e). Similar TLC analysis data were not found in the literature for the enzyme reaction with olive leaf extract. This is the first report of the analysis of olive leaves and onion peel extracts with a TLC plate using ImageJ software (*21*).

### Results of high-performance liquid chromatography analysis

The HPLC analysis of onion peel extract showed a considerable increase in quercetin concentration and significant increase of quercetin 3-glucoside concentration after β-glucosidase treatment (Table 1). The enzyme did not hydrolyze the quercetin 3-glucoside standard (data not shown). Thus, other quercetin glucosides, including quercetin 4-glucoside and quercetin 3,4-di-glucoside were hydrolyzed by β-glucosidase. As a result, the peak obtained with the retention time of 19.65 min in the sample chromatogram before the treatment, related to quercetin 4-glucoside (*9*, *35*), disappeared entirely after the enzymatic hydrolysis (data not shown). Similar results were observed in the mentioned literature for onion waste treated with *Thermotoga neapolitana* β-glucosidase. Moreover, β-glucosidase affected other glycosides in the onion peel extract.

**Table 1:** The results of HPLC analysis of onion peel and olive leaf extracts before and after treatment with β-glucosidase

Extract	*γ*(quercetin)/(mg/mL)	*γ*(quercetin-3-O-glucoside)/(mg/mL)	*γ*(oleuropein)/(mg/mL)
Onion peel before treatment	(0.48±0.04)^a^	(0.0018±0.0001)^a^	n.d.
Onion peel after treatment	(1.262±0.03)^b^	(0.054±0.009)^b^	n.d.
Olive leaves before treatment	n.d.	n.d.	(0.4±0.02)^a^
Olive leaves after treatment	n.d.	n.d.	0^b^

Data are mean value±standard deviation, *N*=3. The same letters in each column mean no significant difference at p<0.05. n.d.=not determined

In olive leaf extract, oleuropein (the main phenolic compound) was hydrolyzed completely by β-glucosidase within 24 h of reaction at 50 °C (Table 1). As a result, the bitterness of the extract disappeared after the enzymatic treatment. Still, the oleuropein aglycone peak did not appear in the sample chromatogram after the treatment (data not shown). The oleuropein aglycon has poor water stability. Typically, 24 h after production, it spontaneously or in the reaction with esterase converts to hydroxytyrosol and elenolic acid. Also, in the treated sample, a peak with a retention time of 5.22 min appeared, related to hydroxytyrosol (*10*, *36*). After the treatment with β-glucosidase, the same peak was observed in the oleuropein standard chromatogram, indicating spontaneous hydrolysis of oleuropein aglycon in the water medium.

Furthermore, the enzyme affected other glycosides in the olive leaf extract. The β-glucosidase from different sources, including *Streptomyces* sp. (*37*), almond (*5*, *38*) and olive (*39*, *40*), has been used for oleuropein hydrolysis.

The results of different methods showed the efficacy of β-glucosidase for processing the mentioned by-products. According to the previous study (*13*), *A. niger* β-glucosidase is a stable enzyme with high storage stability, heat stability, pH stability and stability against organic solvents. Therefore, it is a potential enzyme for food waste processing, and extraction and recovery of valuable bioactive compounds with low costs. Moreover, hydrolysis of glycoside compounds into aglycone form increases their health benefits.

## CONCLUSIONS

This study showed the efficacy of β-glucosidase for the hydrolysis of quercetin glycosides in onion peel extract. Also, oleuropein (bitterness agent) in the olive leaf extract was hydrolyzed completely by β-glucosidase. As a result, the extract bitterness was eliminated by enzymatic treatment. Different methods confirmed that the antioxidant activity in onion peel and olive leaf extracts increased after the enzymatic hydrolysis. Thus, the hydrolysis by β-glucosidase improved the bioactive properties of the mentioned extracts, and consenquently the extracts with more potent antioxidant activity and desired flavour can be used as natural and low-cost antioxidants in the food industry. Moreover, the extraction yield of quercetin and hydroxytyrosol was enhanced by β-glucosidase, which makes it an alternative biological method instead of chemical processes to extract these valuable compounds. Also, the treatment of the onion peel and olive leaf extracts with β-glucosidase provides the possibility of reusing these food wastes. As a result, β-glucosidase from *Aspergillus niger* (as a food-grade and inexpensive microorganism for enzyme production and applicable on an industrial scale), a stable enzyme, has a high potential for by-product processing and extraction of bioactive compounds at a low cost, which is very important for the food industry.
